# Supplementary material for: Flavone and Hydroxyflavones Are Ligands That Bind the Orphan Nuclear Receptor 4A1 (NR4A1)
Source: Int J Mol Sci. 2023 May 2;24(9):8152. doi: 10.3390/ijms24098152 (PMC10179475; doi:10.3390/ijms24098152)
Supplement: Supplementary file 1 [file ijms-24-08152-s001.zip › ijms-2372626-supplementary.pdf]

SUPPLEMENTAL FIGURE

Figure S1: Structures of flavonoids used in this study and their docking scores as determined using Maestro/Schrodinger modeling software

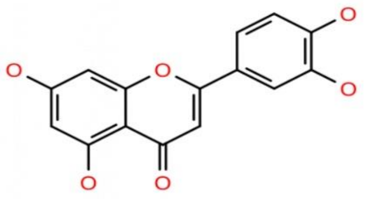

Title: 5,7,3',4'-Tetrahydroxyflavone (Luteolin)
Docking Score: TMZ301/TMZ302 -7.059/-5.832

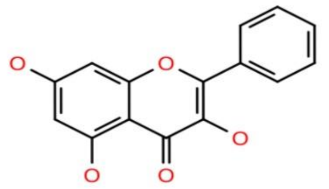

Title: 3,5,7-Trihydroxyflavone (Galangin)
Docking Score: TMZ301/TMZ302 -6.791/-4.83

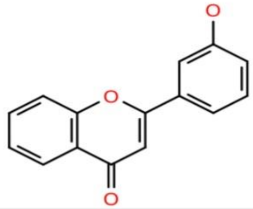

Title: 3'-Hydroxyflavone
Docking Score: TMZ301/TMZ302 -6.756/-5.176

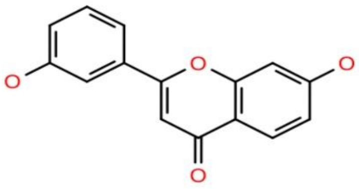

Title: 7,3'-Dihydroxyflavone
Docking Score: TMZ301/TMZ302 -6.719/-5.559

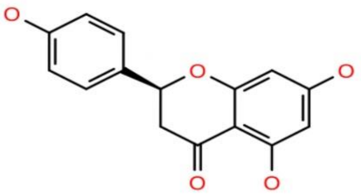

Title: 4',5,7-trihydroxyflavanone (Naringenin)
Docking Score: TMZ301/TMZ302 -6.618/-5.656

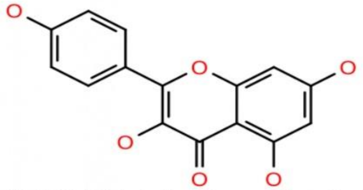

Title:3,5,7,4'-Tetrahydroxyflavone (Kaempferol)
Docking Score: TMZ301/TMZ302 -6.555/-5.433

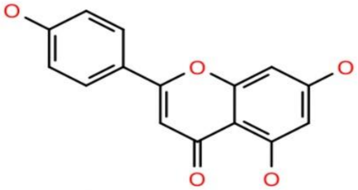

Title: 5,7,4'-Trihydroxyflavone (Apigenin)
Docking Score: TMZ301/TMZ302 -6.441/-4.782

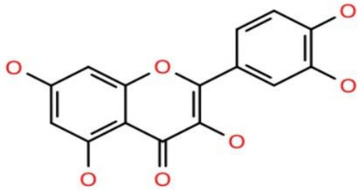

Title:3,7,3',4',5'-Pentahydroxyflavone (Quercetin)
Docking Score: TMZ301/TMZ302 -6.437/-5.397

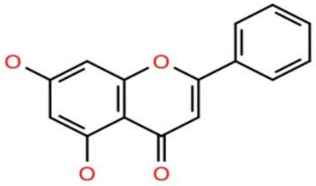

Title: 5,7 Dihydroxyflavone
Docking Score: TMZ301/TMZ302 -6.399/-5.543

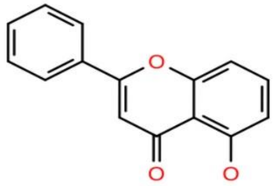

Title: 5-Hydroxyflavone
Docking Score: TMZ301/TMZ302 -6.302/-4.732

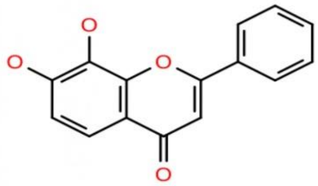

Title: 7,8-Dihydroxyflavone
Docking Score: TMZ301/TMZ302 -5.941/-5.049

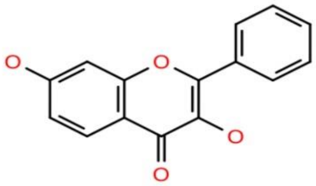

Title: 3,7-Dihydroxyflavone
Docking Score: TMZ301/TMZ302 -5.934/-5.851

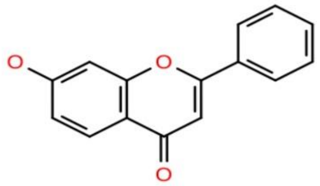

Title: 7-Hydroxyflavone
Docking Score: TMZ301/TMZ302 -5.878/-5.477

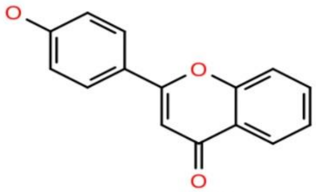

Title: 4'-Hydroxyflavone
Docking Score: TMZ301/TMZ302 -5.861/-4.717

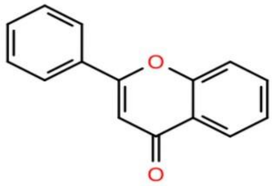

Title: Flavone
Docking Score: TMZ301/TMZ302 -5.741/-4.582

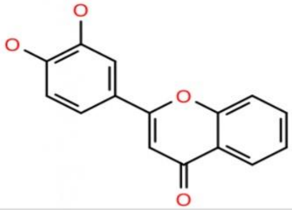

Title: 3',4'-Dihydroxyflavone
Docking Score: TMZ301/TMZ302 -5.736/-5.507

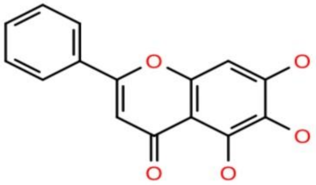

Title: 5,6,7-Trihydroxyflavone (Baicalein)
Docking Score: TMZ301/TMZ302 -5.711/-5.711

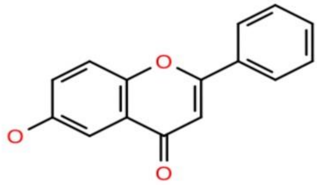

Title: 6-Hydroxyflavone
Docking Score: TMZ301/TMZ302 -5.619/-5.4

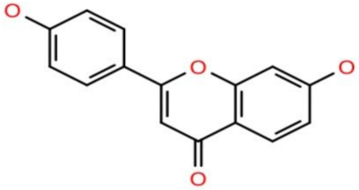

Title: 7,4'-Dihydroxyflavone
Docking Score: TMZ301/TMZ302 -5.575/-5.42

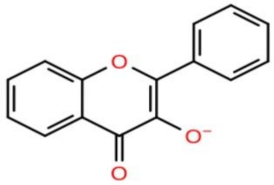

Title: 3-Hydroxyflavone
Docking Score: TMZ301/TMZ302 -5.507/-4.716

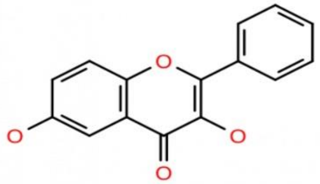

Title: 3,6-Dihydroxyflavone
Docking Score: TMZ301/TMZ302 -4.695/-5.51
